# Analysis of Six Reviews on the Quality of Instruments for the Evaluation of Interprofessional Education in German-Speaking Countries

**DOI:** 10.3205/zma001113

**Published:** 2017-08-15

**Authors:** Jan P. Ehlers, Sylvia Kaap-Fröhlich, Cornelia Mahler, Theresa Scherer, Marion Huber

**Affiliations:** 1 Witten/Herdecke University, Faculty of Health, Chair of Didactics and Educational Research in Health Science, Witten, Germany; 2 University of Zurich, Faculty of Medicine, Dean's Office for Student Affairs, Zurich, Switzerland; 3 University of Heidelberg, Medical Faculty, Department of General Medicine and Health Services, Heidelberg, Germany; 4 Bern University of Applied Sciences, Health Division, Bachelor of Science Programme in Nursing, Bern, Switzerland; 5 Zurich University of Applied Sciences, Interprofessional Teaching, Zurich, Switzerland

**Keywords:** interprofessional education, evaluation, methods, validation, quality criteria

## Abstract

**Background: **More and more institutions worldwide and in German-speaking countries are developing and establishing interprofessional seminars in undergraduate education of health professions. In order to evaluate the different didactic approaches and different outcomes regarding the anticipated interprofessional competencies, it is necessary to apply appropriate instruments. Cross-cultural instruments are particularly helpful for international comparability. The Interprofessional Education working group of the German Medical Association (GMA) aims at identifying existing instruments for the evaluation of interprofessional education in order to make recommendations for German-speaking countries.

**Methods: **Systematic literature research was performed on the websites of international interprofessional organisations (CAIPE, EIPEN, AIPEN), as well as in the PubMed and Cinahl databases. Reviews focusing on quantitative instruments to evaluate competencies according to the modified Kirkpatrick competency levels were searched for. Psychometrics, language/country and setting, in which the instrument was applied, were recorded.

**Results: **Six reviews out of 73 literature research hits were included. A large number of instruments were identified; however, their psychometrics and the applied setting were very heterogeneous. The instruments can mainly be assigned to Kirkpatrick levels 1, 2a & 2b. Most instruments have been developed in English but their psychometrics were not always reported rigorously. Only very few instruments are available in German.

**Conclusion:** It is difficult to find appropriate instruments in German. Internationally, there are different approaches and objectives in the measurement and evaluation of interprofessional competencies. The question arises whether it makes sense to translate existing instruments or to go through the lengthy process of developing new ones.

The evaluation of interprofessional seminars with quantitative instruments remains mainly on Kirkpatrick levels 1 and 2. Levels 3 and 4 can probably only be assessed with qualitative or mixed methods. German language instruments are necessary.

## Zusammenfassung

**Hintergrund: **Sowohl weltweit als auch in den deutschsprachigen Ländern erfolgt zunehmend die Entwicklung und Etablierung von interprofessionellen Lehr- und Lernszenarien in den Ausbildungen der Gesundheitsberufe. Um die unterschiedlichen didaktischen Herangehensweisen wie auch die unterschiedlichen, situationsabhängigen Ergebnisse der Vermittlung interprofessioneller Kompetenzen zu evaluieren, ist es erforderlich, geeignete Instrumente einzusetzen. Dabei sind gerade interkulturelle Instrumente hilfreich, um eine internationale Vergleichbarkeit zu erzielen. Die GMA-Arbeitsgruppe „Interprofessionelle Ausbildung in den Gesundheitsberufen“ hat sich zum Ziel gesetzt, bestehende Instrumente zur Evaluation interprofessioneller Lehre zu identifizieren, um Empfehlungen für den deutschsprachigen Raum auszusprechen.

**Methoden:** Eine systematische Literaturrecherche auf den Internetseiten zur interprofessionellen Ausbildung internationaler Fachorganisationen (CAIPE, EIPEN, AIPEN) sowie in den Datenbanken PubMed und Cinahl wurde durchgeführt. Reviews mit Hauptaugenmerk auf quantitativen Instrumenten zur Evaluation des interprofessionellen Kompetenzerwerbs auf Grundlage der modifizierten Kirkpatrick Kompetenzstufen wurden gesucht. Diese wurden hinsichtlich ihrer psychometrischen Eigenschaften, Sprache/Land und Voraussetzungen des Instrumenteinsatzes untersucht und erfasst.

**Ergebnisse: **Es wurden sechs Reviews aus 73 Treffern der Literatursuche inkludiert. Eine Großzahl an Instrumenten wurde identifiziert, deren Gütekriterien und Einsatz allerdings sehr heterogen war, weshalb sie nicht in die Untersuchung aufgenommen wurden. Die Instrumente sind vorwiegend den Kirkpatrick Stufen 1, 2a & 2b zuzuordnen. Die meisten wurden in englischer Sprache entwickelt. Gütekriterien wurden selten ausführlich berichtet. Nur sehr wenige Instrumente sind in deutscher Sprache verfügbar.

**Schlussfolgerung: **Es ist schwierig geeignete deutschsprachige Instrumente zu finden. International gibt es unterschiedliche Ansätze und Zielsetzungen in der Evaluation und Messung der interprofessionellen Kompetenzen, die sich z.B. nicht allein auf die Ausbildung sondern oft auf das spätere Handeln im Team beziehen. Es stellt sich die Frage, ob es sinnvoll ist, bereits bestehende Instrumente zu übersetzen oder den langen Prozess der eigenen Entwicklung zu verfolgen.

Die Evaluation von interprofessioneller Lehre mit quantitativen Instrumenten erfolgt bisher vorwiegend auf den Stufen 1 und 2. Die Stufen 3 und 4 können größtenteils wahrscheinlich nur mit qualitativen Methoden oder “mixed methods” erfasst werden. Neben dem auf deutsch vorliegendem UWEIPQ ist die Entwicklung weiterer Instrumente in deutscher Sprache oder die Lokalisierung (Übersetzung und Anpassung an nationale Gegebenheiten) englischer Instrumente (z.B. ISVS) notwendig.

## 1. Introduction

Interprofessional collaboration in health care is growing in importance in German-speaking countries. At the same time, more and more institutions are establishing interprofessional teaching and learning scenarios in the education of health professions^1^. 

### 1.1. Background to the Evaluation of Interprofessional Education 

With a history of merely 25 years, interprofessional education is a relatively new field in teaching and affiliated research using different teaching and learning approaches with respect to methodology (e.g. joint seminars or project work), seminar and course composition (e.g. different health professions with and without physicians) and content (e.g. understanding one’s role, team building or professional skills). The special edition of the GMS Journal for Medical Education [12] provides an excellent overview of projects in German-speaking countries. Systematic reviews on the evaluation of interprofessional education have shown positive effects on education as well as on the collaboration between different health professions [8], [17]. However, the studies‘ heterogeneity [8], a missing theoretical, competence-oriented interprofessional education framework [17] as well as missing comparable and standardized evaluation instruments posed a problem for general interpretation of the studies included in the reviews. Alongside different interprofessional competency frameworks have therefore been developed in recent years [4], [7], [11], [15]. Moreover, recommendations for planning and performing evaluation studies have been established [16] and various evaluation instruments developed and tested in different settings [3]. 

Prior to applying instruments, it needs to be decided which instrument is suitable for which learning outcome. In this respect, the Kirkpatrick four-level framework for evaluating the results of educational programmes serves as orientation. For the evaluation of interprofessional education [1], [5], the original four competence levels were developed further into six levels (see Table 1 (Tab. 1)). In doing so, the original levels 2 “Learning” and 4 “Results” were each divided into two levels in order to distinguish learning results on the personal and the health-care level. Despite the fact that the model has been criticized and despite its limitations [20] it provides a basic and clear framework. In the meantime, the six levels have been frequently applied when evaluating educational measures in health professional training [20]. These levels therefore provide a good orientation for a practice-oriented selection of comparable evaluation instruments. 

#### 1.2. Research Objective

In order to be able to assess interprofessional teaching quality, it is necessary to use reliable and valid instruments. In a first step, existing instruments were to be identified in the literature and assessed regarding their basic, but also specific suitability for use in the German-speaking countries. Due to the manifold and heterogeneous publications in this field, it was decided not to consult individual studies but rather reviews. 

 Identified suitable instruments will then be made available for German-speaking countries. Based on the results, a practice-oriented handout with instruments for the evaluation of interprofessional seminars and projects shall be provided in the years to come. 

The following research questions were derived from these considerations: Which instruments are recommended in the summarized review literature for the evaluation of interprofessional teaching on which Kirkpatrick evaluation level?

Which instruments are available in German and which can be recommended for translation into German respectively?

## 2. Method

A two phase methodological approach was necessary. 

### First Phase

As the field of interprofessional collaboration and teaching is developing rapidly, the websites of the relevant national and international interprofessional networks (CAIPE - Centre for the Advancement of Interprofessional Education, EIPEN - European Interprofessional Practice and Education Network, AIPEN - Australasian Interprofessional Practice and Education Network, NIPNET - Nordic Interprofessional Network) were scanned systematically for instruments for the evaluation of teaching activities.

#### Second Phase

In order to further address the research questions, the aim was to compile a systematic literature review. As mentioned in the Research Objective section, this systematic review was not to be based on individual studies, but rather on a meta-synthesis of reviews. The main focus was on review articles of instruments for evaluation as assessment tool, not for evaluation as complete control cycle. The evaluation tools were to be suitable for use on the teaching level, not on the collaboration level. 

PubMed/Medline and CINAHL were identified as suitable literature databases. Database-specific search strategies were determined, including the search keys “review”, “evaluation” and “interprofessional education” (see Attachment 1 for detailed search strategies). In a next step, publications found twice or being unsuitable as regards content were consensually eliminated by three experts of the working group. Only reviews were included which:

- Consult studies on evaluation instruments for interprofessional education
- Assign them to evaluation levels according to Kirkpatrick [5] and
- Describe the psychometric quality criteria.

Afterwards, two experts (MH, JE) independently checked the reviews being determined this way and included in the study by means of criteria stipulated in the Critical Appraisal Skills Programme Checklist (CASP; [19]). The reviews were checked for quality based on the quality of the research objective, literature research, publications, quality control and results. First, the experts filled in the checklist independently, then compared the results and after checking contentious reviews again agreed consensually on the reviews to be included. 

Two independent working group experts (MH, JE) subsequently assessed the information on the test quality of the individual studies in the respective reviews. A brief characterization describes the suitable instruments determined this way. An adequate test quality description contains comprehensive details on objectivity, reliability, validity and on diagnostic psychometric properties (such as sensitivity and specificity).

Particularly successful tools as well as German-language tools were individually and critically discussed again and presented.

## 3. Results

### First Phase

The scanning of the interprofessional networks yielded two repositories for evaluation tools:

- Canadian Interprofessional Health Collaborative [15]
- National Center for Interprofessional Practice and Education [3].

Both collections comprehensively present tools, but also show limitations. They do not only focus on interprofessional education, but also consider instruments for evaluating interprofessional collaboration. The access to these databases is rather open, enabling a free download of tools. However, the integrated tools are not subject to critical evaluation and quality management, rendering their validity and reliability uncertain. The review at hand therefore does not include any tools of these databases. 

#### Second Phase

**Figure 1:**
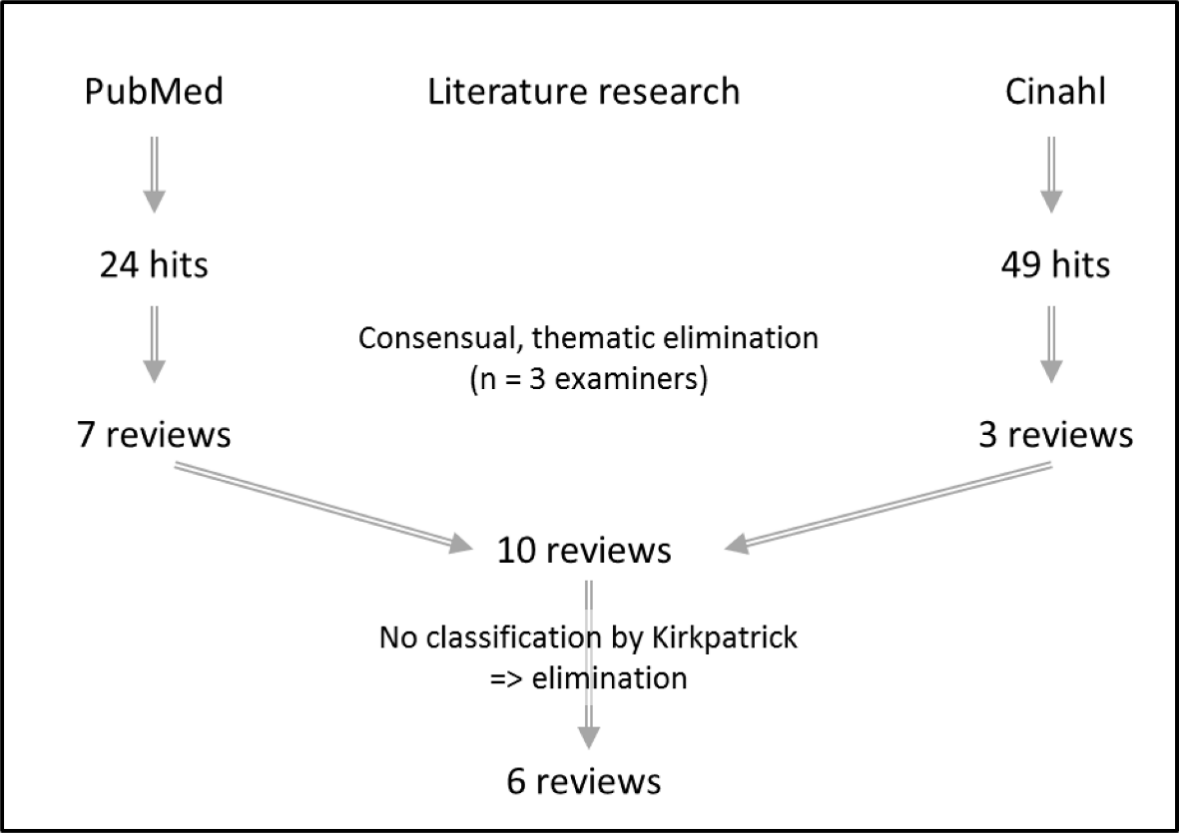
Flowchart demonstrating results of the literature search

The PubMed search produced 24 hits while the Cinahl search produced 49 hits (see Attachment 1 and Figure 1 (Fig. 1)). After scanning the abstracts, 63 publications could be excluded as they did not deal with the sought-for topic or did not comply with the requirements agreed upon (e.g. interprofessional collaboration or qualitative methods). Out of the ten suitable reviews, the studies of four reviews were not assigned to the Kirkpatrick levels [5]. 

**Figure 2:**
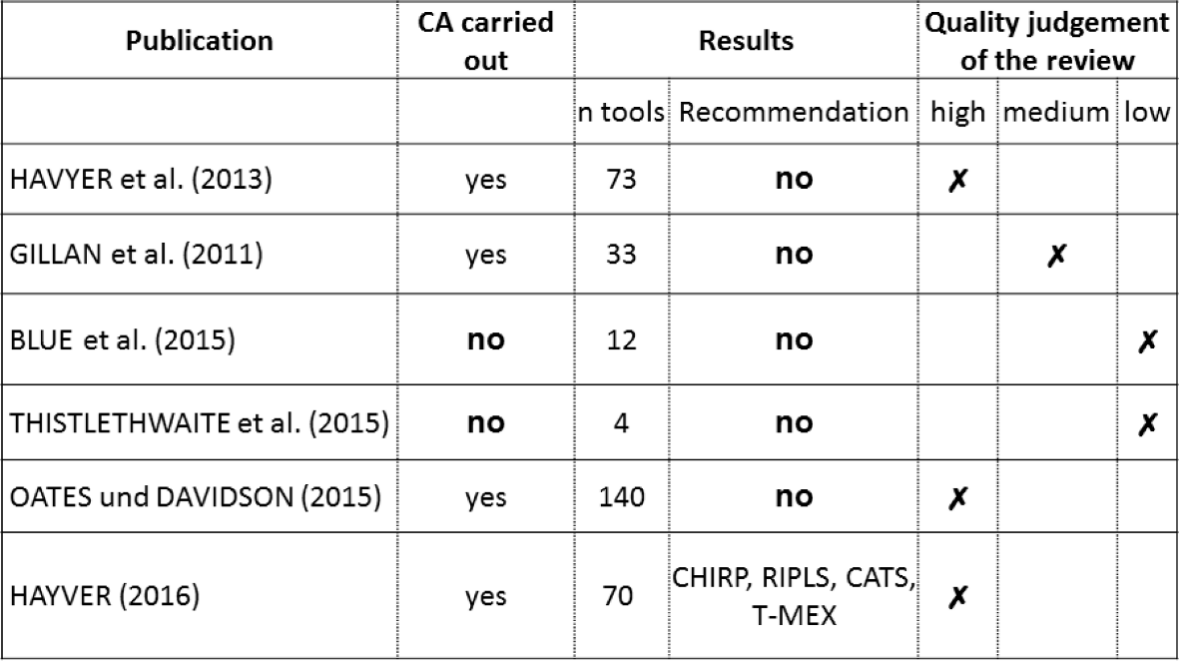
Overview of the consensual evaluation of six review articles by two examiners (CA=Critical Appraisal)

The working group assessed the six remaining reviews using the CASP checklist and went through the full text in search of valid and suitable tools (see Table 2 (Tab. 2) and Figure 2 (Fig. 2)). 

The evaluation of the reviews shows different results regarding the quality of the included reviews and also regarding the recommended measuring instruments. Three reviews are high-quality reviews [9], [10], [14]. All three reviews are based on standards (e.g. Cochrane Guidelines for Systematical Reviews or Standards for evaluating the assessment tools included in the studies). Furthermore, these three reviews clearly assign identified evaluation tools to the Kirkpatrick levels. The review at hand also includes a medium-quality review [6] and two low-quality reviews [2], [20]. These three reviews primarily either performed an exploratory search or the literature review was part of a multi-method survey. The fact that the studies included in these reviews were not critically examined significantly reduces the validity of the reviews. Moreover, an evaluation of the assessments regarding their psychometric properties took place only partly or not at all. Cronbach’s Alpha is the value most mentioned in this context. There was no information on further psychometric properties available. 

Havyer et al. [9] are the only ones to make a clear recommendation based on the test quality criteria. From their point of view, the Collaborative Healthcare Interdisciplinary Relationship Planning (CHRIP), the Readiness for Interprofessional Education Scale (RIPLS), the Communication and Teamwork Skills assessment (CATS) and the Teamwork Mini-Clinical Evaluation Exercise (T-MAX) tools meet the quality criteria. Their 2014 publication does expressly state but not explicitly recommend the Safety Attitudes Questionnaire (SAQ) and the Team Climate Inventory (TCI). Recommendations of Havyer et al. [10] refer to a specific competency framework of the Association of American Medical Colleges (AAMC). Cronbach’s Alpha as a measure for reliability was the only indicator for quality criteria mostly stated by Havyer et al. [9], [10] for the included studies. They did not report on factor-analytical findings for indicating content validity. 

After having determined own minimum test theory quality criteria, Oates and Davidson [14] conclude that only the Interprofessional Socialization and Valuing Scale (ISVS) meets all minimum quality standards. Five further instruments (Interprofessional Collaborator Assessment Rubric - ICAR, Attitudes Towards Teamwork in Training Undergoing Designed Educational Simulation - KidSIM ATTITUDES Questionnaire, Interdisciplinary Education Perception Scale - IEPS and University of the West of England Interprofessional Questionnaire - UWEIPQ) only partly comply with the minimum standards. Three instruments (RIPLS, Attitudes to Shared Learning - ASL and StudDat Questionnaire) do either not meet the standards at all or just insufficiently.

Gillan et al. [6] offer a comprehensive overview of evaluation instruments and categorize all items from the found instruments. As the literature research was described in detail, this review can be regarded as medium-quality review. The clear assignment of the items contained in the evaluation tools to the Kirkpatrick levels [5] is particularly useful in practice. The authors therefore offer a very transparent overview and show that an assignment to levels 1 to 4a is possible. No item could be assigned to level 4b though. 

Blue et al. [2] exclusively included reviews in their article. However, their description of the kinds of reviews they included in their review article is imprecise. A critical assessment of the included reviews is non-existent. The exploratory literature research, however, was performed as addition to a "mixed method" survey, the content of which does not refer to the present research objective. Therefore, only the review part of Blue et al. [2] was reviewed for quality. 

Thistlethwaite et al. [20] were primarily looking for intervention studies containing instruments for evaluating interprofessional education and collaboration in their review. It should be noted that the authors searched directly in journals only and did not scan any databases. Moreover, the search keys of this literature research are not evident. Thistlethwaite et al. [20] recommend a multi-method survey of interprofessional competencies. 

The overall conclusion is that a wide range of instruments has been developed, which are applied on different levels of the modified Kirkpatrick framework [2]. The scanning of full texts showed that presumably there is no quantitative evaluation instrument for level 4b [1]. 

Furthermore, validation and quality criteria, evidence level and evaluation levels according to the modified Kirkpatrick framework [1] were very heterogeneous.

Nevertheless, the following four instruments meeting the search requirements, i.e. assignment to one or more Kirkpatrick levels and/or interprofessional competencies, could be determined: Readiness for Interprofessional Learning Scale (RIPLS), University of the West of England Interprofessional Questionnaire (UWEIPQ), Interprofessional Socialization and Valuing Scale (ISVS) and Team Climate Inventory (TCI). Attachment 2 contains a brief description of the individual instruments.

## 4. Discussion

The already existing online repositories of tools for evaluating interprofessionalism have shown that it is inevitable to focus on specific questions and quality assurance regarding usability and transparency. The present review reveals that the unsystematic approach in the scanned reviews and thus the missing critical assessment of the studies included in the reviews proved to be problematic. 

In conclusion, the heterogeneous situation regarding the quality of the reviews included in this study only allows for a limited recommendation. From the test theory perspective, the recommendation of Havyer et al. [9] should be considered. However, as it is not clearly evident how the recommendation came about, it should be treated with caution as well because in particular the use of RIPLS has been critically discussed in the meantime due to its quality criteria [13], [18]. None of the reviews mentions psychometric measures such as sensitivity or specificity regarding test methods. Moreover, construct validity verification has not been described for any of the mentioned instruments. The review only in part reports that a factor analysis was performed. The specific construct to be reviewed by means of the tools can only be measured content-wise but is not verifiable by means of test theory methods. A review of different evaluation instruments regarding concurrent validity would be beneficial at this point. Information on objectivity and inter rater reliability, respectively, is missing as well, but would be important with respect to the test quality. The fact that most of the time the authors only report on the instrument reliability indicates a lack of theoretical orientation towards a framework when developing the instruments. This orientation, however, would enable a validity check. 

Various instruments are available measuring interprofessional education by evaluating outcomes on different Kirkpatrick levels. Outcome and Kirkpatrick level have to be considered when selecting or applying an evaluation instrument. Only a few reliable and valid instruments are available yet. Assigning an outcome to a competency framework may be helpful when choosing an instrument.

Moreover, not all Kirkpatrick levels can be mapped by quantitative assessments. It seems to be necessary to include qualitative evaluation as well.

TCI and ISVS are mentioned in various reviews. Oates and Davidson [14] describe the ISVS as the only instrument meeting the test quality criteria. Moreover, the ISVS can be assigned to three Kirkpatrick levels and is also mentioned as instrument in all other reviews. The RIPLS is certainly considered as one of the instruments being applied most and it would also be available in German. However, it is not recommendable from a test theory perspective, as critically commented by Oates and Davidson [14] as well. The UWEIPQ is also an often-used instrument and available in German, but only partly meets the quality criteria. 

Finally, the ISVS is the only instrument to be recommended from a test theoretical perspective at the moment. As this high-quality tool is already available and does not need to be redeveloped, the working group is currently translating and localizing the ISVS. It will also validate the German version. 

## 5. Conclusion

The results of this review show that the transition from education to practice also needs to come more into focus. This includes evaluations on team level, an analysis of the transition and the development of suitable instruments and benchmarks for ensuring business success and organizational modifications (e.g. patient safety, job satisfaction, critical appraisal).

Another working group of the GMA Committee for Interprofessional Education is currently working on a German competency framework for interprofessional education. After the compilation and approval of this competency framework, the suitability of the recommended instruments shall be reviewed again. Afterwards, it can be decided on whether renewed, adapted recommendations or new instruments appropriate to the complexity shall be developed. 

## Notes

^1^ In this context, health professions comprise professions in the fields of medicine, dentistry, nursing, therapy and diagnostics.

## Competing interests

The authors declare that they have no competing interests.

## Supplementary Material

Strategies, search terms and results of the literature research

Short presentation of individual instruments

## Figures and Tables

**Table 1:**
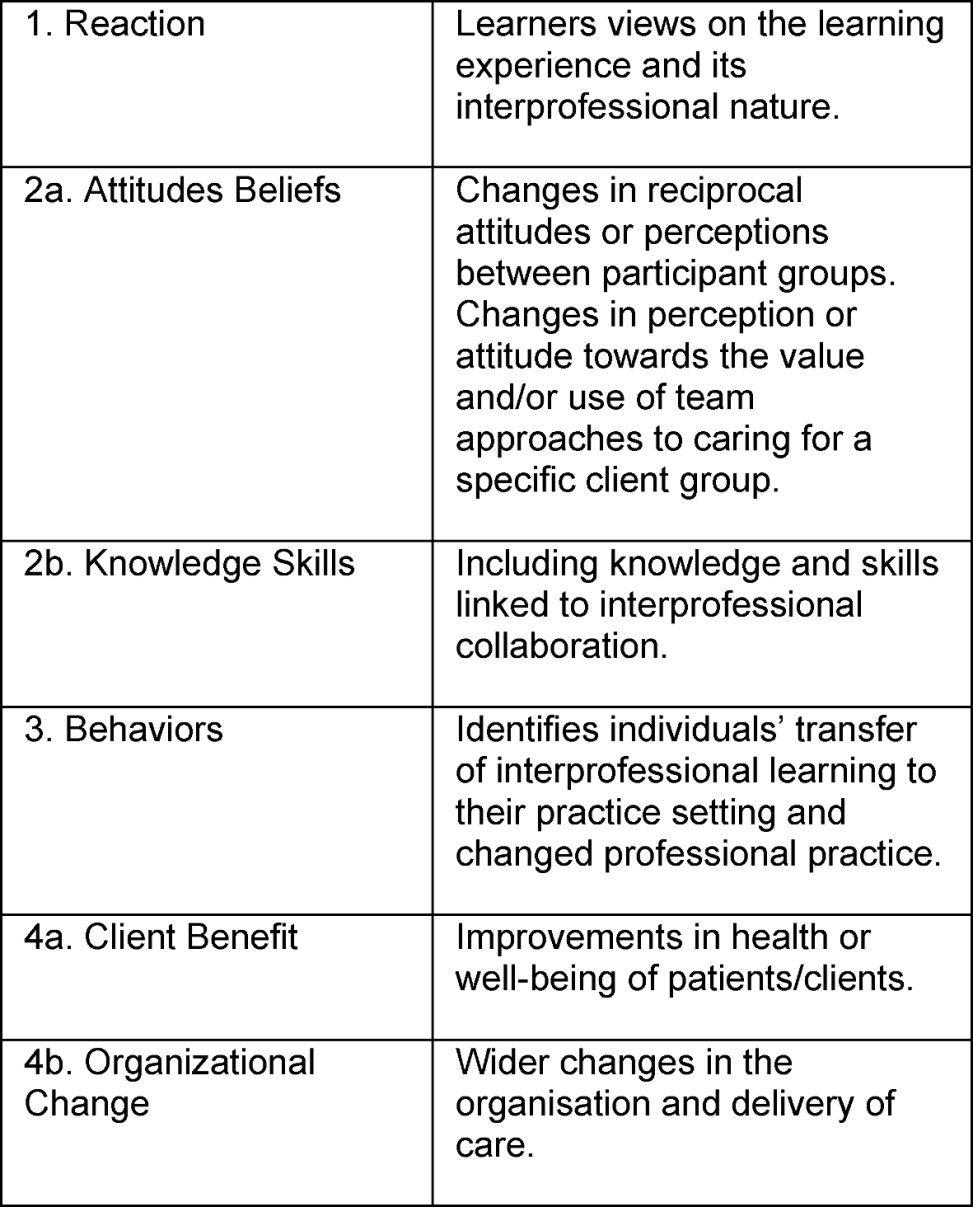
Kirkpatrick’s competence levels modified for the evaluation of interprofessional education [1]

**Table 2:**
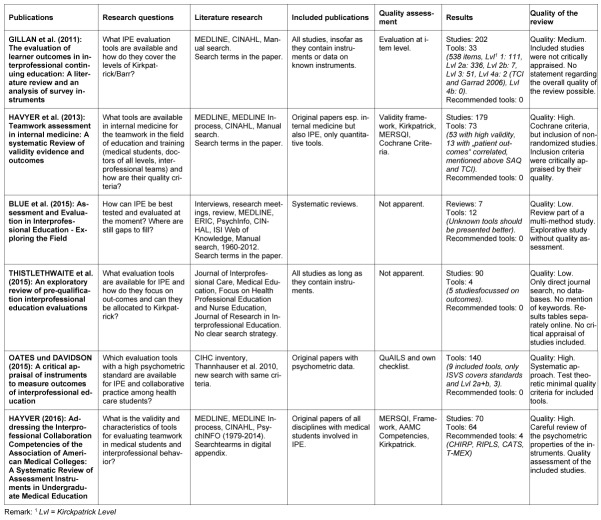
Six review articles about the quality of evaluation tools for interprofessional education rated by CASP (Critical Appraisal Skills Programme Checklist)

## References

[1] Barr H, Freeth D, Hammick M, Koppel I, Reeves S (2000). Evaluations of interprofessional education.

[2] Blue AV, Chesluk BJ, Conforti LN, Holmboe ES (2015). Assessment and Evaluation in Interprofessional Education: Exploring the Field. J Allied Health.

[3] Brandt BF (2014). Update on the US national center for interprofessional practice and education. J Interprof Care.

[4] Brewer M (2011). Curtin University's faculty of health sciences interprofessional capability framework.

[5] Freeth D, Hammick M, Koppel I, Reeves S, Barr H (2002). A critical review of evaluations of interprofessional education.

[6] Gillan C, Lovrics E, Halpern E, Wiljer D, Harnett N (2011). The evaluation of learner outcomes in interprofessional continuing education: a literature review and an analysis of survey instruments. Med Teach.

[7] Gordon F (2006). Combined Universities Interprofessional Learning Unit: Final Report.

[8] Hammick M, Freeth D, Koppel I, Reeves S, Barr H (2007). A best evidence systematic review of interprofessional education: BEME Guide no. 9. Med Teach.

[9] Havyer RD, Nelson DR, Wingo MT, Comfere NI, Halvorsen AJ, McDonald FS, Reed DA (2016). Addressing the Interprofessional Collaboration Competencies of the Association of American Medical Colleges: A Systematic Review of Assessment Instruments in Undergraduate Medical Education. Acad Med.

[10] Havyer RD, Wingo MT, Comfere NI, Nelson DR, Halvorsen AJ, McDonald FS, Reed DA (2014). Teamwork assessment in internal medicine: a systematic review of validity evidence and outcomes. J Gen Intern Med.

[11] Interprofessional Education Collaborative Expert Panel (2011). Core competencies for interprofessional collaborative practice.

[12] Klapper B, Schirlo C (2016). Special edition booklet: Interprofessional Training – Published by the Robert Bosch Stiftung and the Gesellschaft für Medizinische Ausbildung. GMS J Med Educ.

[13] Mahler C, Berger S, Pollard K, Krisam J, Karstens S, Szecsenyi J, Krug K (2016). Translation and psychometric properties of the German version of the University of the West of England Interprofessional Questionnaire (UWE-IP). J Interprof Care.

[14] Oates M, Davidson M (2015). A critical appraisal of instruments to measure outcomes of interprofessional education. Med Educ.

[15] Orchard C, Bainbridge L, Bassendowski S, Stevenson K, Wagner SJ, Weinberg L, Curran V, Di Loreto L, Sawatsky-Girling B (2010). A national interprofessional competency framework.

[16] Reeves S, Boet S, Zierler B, Kitto S (2015). Interprofessional Education and Practice Guide No. 3: Evaluating interprofessional education. J Interprof Care.

[17] Reeves S, Goldman J, Burton A, Sawatzky-Girling B (2010). Synthesis of systematic review evidence of interprofessional education. J All Health.

[18] Schmitz CC, Brandt BF (2015). The Readiness for Interprofessional Learning Scale: To RIPLS or not to RIPLS? That is only part of the question. J Interprof Care.

[19] Singh J (2013). Critical appraisal skills programme. J Pharmacol Pharmacother.

[20] Thistlethwaite J, Kumar K, Moran M, Saunders R, Carr S (2015). An exploratory review of pre-qualification interprofessional education evaluations. J Interprof Care.

