# Supplementary material for: Analysis of Six Reviews on the Quality of Instruments for the Evaluation of Interprofessional Education in German-Speaking Countries
Source: GMS J Med Educ. 2017 Aug 15;34(3):Doc36. doi: 10.3205/zma001113 (PMC5569985; doi:10.3205/zma001113)
Supplement: Strategies, search terms and results of the literature research [file JME-34-36-s-001.pdf]

Pubmed/Medline-research was performed on 04/18/2916 with the following search term:

("review"[Publication Type] OR "review literature as topic"[MeSH Terms] OR "review"[All Fields]) AND interprofessional[All Fields] AND ("education"[Subheading] OR "education"[All Fields] OR "educational status"[MeSH Terms] OR ("educational"[All Fields] AND "status"[All Fields]) OR "educational status"[All Fields] OR "education"[All Fields] OR "education"[MeSH Terms]) AND ("evaluation studies"[Publication Type] OR "evaluation studies as topic"[MeSH Terms] OR "evaluation"[All Fields]) AND tools[All Fields])

**Hits:** 24 (7 matches)

(Armbruster, 2002, Mackay, 2004, Veloski et al., 2005, Kalb et al., 2006, Davidson et al., 2008, Jones et al., 2008, Parker, 2009, Buljac-Samardzic et al., 2010, Harrison & Fopma-Loy, 2010, Van Nortwick et al., 2010, George, 2011, Gillan et al., 2011, Fisher & King, 2013, Grand et al., 2013, Murdoch et al., 2013, Havyer et al., 2014, Marrocco, 2014, Niebuhr et al., 2014, Reynolds et al., 2014, Blue et al., 2015, Frantz et al., 2015, Havyer et al., 2015, Thistlethwaite et al., 2015, Korner et al., 2016)

Matches:

BLUE, A. V., CHESLUK, B. J., CONFORTI, L. N. & HOLMBOE, E. S. 2015. Assessment and evaluation in interprofessional education: exploring the field. *J Allied Health,* 44**,** 73-82.

GILLAN, C., LOVRICS, E., HALPERN, E., WILJER, D. & HARNETT, N. 2011. The evaluation of learner outcomes in interprofessional continuing education: a literature review and an analysis of survey instruments. *Med Teach,* 33**,** e461-70.

HAVYER, R. D., NELSON, D. R., WINGO, M. T., COMFERE, N. I., HALVORSEN, A. J., MCDONALD, F. S. & REED, D. A. 2015. Addressing the Interprofessional Collaboration Competencies of the Association of American Medical Colleges: A Systematic Review of Assessment Instruments in Undergraduate Medical Education. *Acad Med*. Epub ahead

HAVYER, R. D., WINGO, M. T., COMFERE, N. I., NELSON, D. R., HALVORSEN, A. J., MCDONALD, F. S. & REED, D. A. 2014. Teamwork assessment in internal medicine: a systematic review of validity evidence and outcomes. *J Gen Intern Med,* 29**,** 894-910.

KORNER, M., BUTOF, S., MULLER, C., ZIMMERMANN, L., BECKER, S. & BENGEL, J. 2016. Interprofessional teamwork and team interventions in chronic care: A systematic review. *J Interprof Care,* 30**,** 15-28.

MACKAY, S. 2004. The role perception questionnaire (RPQ): a tool for assessing undergraduate students' perceptions of the role of other professions. *J Interprof Care,* 18**,** 289-302.

THISTLETHWAITE, J., KUMAR, K., MORAN, M., SAUNDERS, R. & CARR, S. 2015. An exploratory review of pre-qualification interprofessional education evaluations. *J Interprof Care,* 29**,** 292-7.

Cinahl research was performed on 02/28/2916 with the following search term:

(review or overview or meta-analysis) AND interprofessional education AND evaluation
Narrow by SubjectMajor: - education, interdisciplinary

**Hits:** 49 (3 matches)

(Hall & Weaver, 2001, Koppel et al., 2001, MacDougall et al., 2001, McNair et al., 2001, Reeves, 2001, Cullen et al., 2003, Priest et al., 2005, Clemow, 2007, Ireland et al., 2008, O'Brien et al., 2008, Wright & Lindqvist, 2008, Conway, 2009, Goldman et al., 2009, Hegmann et al., 2009, Pulman et al., 2009, Reeves, 2009, Greer & Clay, 2010, Reeves et al., 2010, Thannhauser et al., 2010, Thistlethwaite & Moran, 2010, Wilson et al., 2010, Laurenson & Brocklehurst, 2011, Reeves et al., 2011, Waterston, 2011, Zhang et al., 2011, Abu-Rish et al., 2012, Gough et al., 2012, Thistlethwaite, 2012, VanLeit et al., 2012, Barr, 2013, George, 2013, King et al., 2013, Kitto et al., 2013, Kvan, 2013, Newton et al., 2013, Suter et al., 2013, Anderson et al., 2014, Brandt et al., 2014, Courtenay et al., 2014a, Courtenay et al., 2014b, Hoti et al., 2014, Lawlis et al., 2014, Menard & Varpio, 2014, Shoemaker et al., 2014, Sockalingam et al., 2014, Kent & Keating, 2015, Oates & Davidson, 2015, Solberg et al., 2015, O'Carroll et al., 2016)

Matches:

OATES, M. & DAVIDSON, M. 2015. A critical appraisal of instruments to measure outcomes of interprofessional education. *Medical Education,* 49**,** 386-398.

THANNHAUSER, J., RUSSELL-MAYHEW, S. & SCOTT, C. 2010. Measures of interprofessional education and collaboration. *Journal of Interprofessional Care,* 24**,** 336-349.

THISTLETHWAITE, J. & MORAN, M. 2010. Learning outcomes for interprofessional education (IPE): Literature review and synthesis. *Journal of Interprofessional Care,* 24**,** 503-513.

All hits:


ABU-RISH, E., KIM, S., CHOE, L., VARPIO, L., MALIK, E., WHITE, A. A., CRADDICK, K., BLONDON, K., ROBINS, L., NAGASAWA, P., THIGPEN, A., CHEN, L.-L., RICH, J. & ZIERLER, B. 2012. Current trends in interprofessional education of health sciences students: A literature review. *Journal of Interprofessional Care,* 26**,** 444-451.

ANDERSON, A., CANT, R. & HOOD, K. 2014. Measuring students perceptions of interprofessional clinical placements: Development of the Interprofessional Clinical Placement Learning Environment Inventory. *Nurse Education in Practice,* 14**,** 518-524.

ARMBRUSTER, P. 2002. The administration of school-based mental health services. *Child Adolesc Psychiatr Clin N Am,* 11**,** 23-41.

BARR, H. 2013. Toward a theoretical framework for interprofessional education. *Journal of Interprofessional Care,* 27**,** 4-9.

BLUE, A. V., CHESLUK, B. J., CONFORTI, L. N. & HOLMBOE, E. S. 2015. Assessment and evaluation in interprofessional education: exploring the field. *J Allied Health,* 44**,** 73-82.

BRANDT, B., LUTFIYYA, M. N., KING, J. A. & CHIORESO, C. 2014. A scoping review of interprofessional collaborative practice and education using the lens of the Triple Aim. *Journal of Interprofessional Care,* 28**,** 393-399.

BULJAC-SAMARDZIC, M., DEKKER-VAN DOORN, C. M., VAN WIJNGAARDEN, J. D. & VAN WIJK, K. P. 2010. Interventions to improve team effectiveness: a systematic review. *Health Policy,* 94**,** 183-95.

CLEMOW, R. 2007. An illuminative evaluation of skills rehearsal in a mentorship course. *Nurse Education Today,* 27**,** 80-87.

CONWAY, J. 2009. Implementing interprofessional learning in clinical education: findings from a utility-led evaluation. *Contemporary Nurse: A Journal for the Australian Nursing Profession,* 32**,** 187-200.

COURTENAY, M., BAIR, A., BAKERJIAN, D., EIDSON, S., MURRAY-GARCIA, J., HERBERT, P., HIMMERICK, K., MONGOVEN, J., ROBINSON, M. & WARD, D. 2014a. Interprofessional education: an overview of six initiatives across the schools of health at a single university. *Journal of Interprofessional Care,* 28**,** 155-156.

COURTENAY, M., CONRAD, P., WILKES, M., LA RAGIONE, R. & FITZPATRICK, N. 2014b. Interprofessional initiatives between the human health professions and veterinary medical students: a scoping review. *Journal of Interprofessional Care,* 28**,** 323-330.

CULLEN, L., FRASER, D. & SYMONDS, I. 2003. Strategies for interprofessional education: the Interprofessional Team Objective Structured Clinical Examination for midwifery and medical students. *Nurse Education Today,* 23**,** 427-433.

DAVIDSON, M., SMITH, R. A., DODD, K. J., SMITH, J. S. & O'LOUGHLAN, M. J. 2008. Interprofessional pre-qualification clinical education: a systematic review. *Aust Health Rev,* 32**,** 111-20.

FISHER, D. & KING, L. 2013. An integrative literature review on preparing nursing students through simulation to recognize and respond to the deteriorating patient. *J Adv Nurs,* 69**,** 2375-88.

FRANTZ, J. M., BEZUIDENHOUT, J., BURCH, V. C., MTHEMBU, S., ROWE, M., TAN, C., VAN WYK, J. & VAN HEERDEN, B. 2015. The impact of a faculty development programme for health professions educators in sub-Saharan Africa: an archival study. *BMC Med Educ,* 15**,** 28.

GEORGE, D. R. 2011. "Friending Facebook?" A minicourse on the use of social media by health professionals. *J Contin Educ Health Prof,* 31**,** 215-9.

GEORGE, M. J. 2013. *Transforming Ways of Knowing about Interprofessional Education: A Single Exploratory Case Study with Nursing Educators.* Ed.D., University of Calgary (Canada).

GILLAN, C., LOVRICS, E., HALPERN, E., WILJER, D. & HARNETT, N. 2011. The evaluation of learner outcomes in interprofessional continuing education: a literature review and an analysis of survey instruments. *Med Teach,* 33**,** e461-70.
GOLDMAN, J., ZWARENSTEIN, M., BHATTACHARYYA, O. & REEVES, S. 2009. Improving the clarity of the interprofessional field: implications for research and continuing interprofessional education. *Journal of Continuing Education in the Health Professions,* 29**,** 151-156.
GOUGH, S., HELLABY, M., JONES, N. & MACKINNON, R. 2012. A review of undergraduate interprofessional simulation-based education (IPSE). *Collegian,* 19**,** 153-170.
GRAND, J. A., PEARCE, M., RENCH, T. A., CHAO, G. T., FERNANDEZ, R. & KOZLOWSKI, S. W. 2013. Going DEEP: guidelines for building simulation-based team assessments. *BMJ Qual Saf,* 22**,** 436-48.
GREER, A. G. & CLAY, M. C. 2010. Interprofessional Education Assessment and Planning Instrument for Academic Institutions. *Journal of Allied Health,* 39**,** 224-231.
HALL, P. & WEAVER, L. 2001. Interdisciplinary education and teamwork: a long and winding road. *Medical Education,* 35**,** 867-875.
HARRISON, P. A. & FOPMA-LOY, J. L. 2010. Reflective journal prompts: a vehicle for stimulating emotional competence in nursing. *J Nurs Educ,* 49**,** 644-52.
HAVYER, R. D., NELSON, D. R., WINGO, M. T., COMFERE, N. I., HALVORSEN, A. J., MCDONALD, F. S. & REED, D. A. 2015. Addressing the Interprofessional Collaboration Competencies of the Association of American Medical Colleges: A Systematic Review of Assessment Instruments in Undergraduate Medical Education. *Acad Med*.
HAVYER, R. D., WINGO, M. T., COMFERE, N. I., NELSON, D. R., HALVORSEN, A. J., MCDONALD, F. S. & REED, D. A. 2014. Teamwork assessment in internal medicine: a systematic review of validity evidence and outcomes. *J Gen Intern Med,* 29**,** 894-910.
HEGMANN, T. E., KASSON, B. G. & STAFFORD, H. A. 2009. Comparison of medical and physician assistant student performance in interprofessional pharmacology and clinical medicine courses. *Journal of Physician Assistant Education (Physician Assistant Education Association),* 20**,** 21-25.
HOTI, K., FORMAN, D. & HUGHES, J. 2014. Evaluating an interprofessional disease state and medication management review model. *Journal of Interprofessional Care,* 28**,** 168-170.
IRELAND, J., GIBB, S. & WEST, B. 2008. Interprofessional education: reviewing the evidence. *British Journal of Midwifery,* 16**,** 446-453.
JONES, M., O'CARROLL, P., THOMPSON, J. & D'AMBROSIO, L. 2008. Assessing regional public health preparedness: a new tool for considering cross-border issues. *J Public Health Manag Pract,* 14**,** E15-22.
KALB, K. B., CHERRY, N. M., KAUZLORIC, J., BRENDER, A., GREEN, K., MIYAGAWA, L. & SHINODA-METTLER, A. 2006. A competency-based approach to public health nursing performance appraisal. *Public Health Nurs,* 23**,** 115-38.
KENT, F. & KEATING, J. L. 2015. Interprofessional education in primary health care for entry level students — A systematic literature review. *Nurse Education Today,* 35**,** 1221-1231.
KING, S., DRUMMOND, J., HUGHES, E., BOOKHALTER, S., HUFFMAN, D. & ANSELL, D. 2013. An inter-institutional collaboration: transforming education through interprofessional simulations. *Journal of Interprofessional Care,* 27**,** 429-431.
KITTO, S., NORDQUIST, J., PELLER, J., GRANT, R. & REEVES, S. 2013. The disconnections between space, place and learning in interprofessional education: an overview of key issues. *Journal of Interprofessional Care,* 27**,** 5-8.
KOPPEL, I., BARR, H., REEVES, S., FREETH, D. & HAMMICK, M. 2001. Establishing a systematic approach to evaluating the effectiveness of interprofessional education. *Issues in Interdisciplinary Care,* 3**,** 41-49.

KORNER, M., BUTOF, S., MULLER, C., ZIMMERMANN, L., BECKER, S. & BENGEL, J. 2016. Interprofessional teamwork and team interventions in chronic care: A systematic review. *J Interprof Care,* 30**,** 15-28.
KVAN, T. 2013. Evaluating learning environments for interprofessional care. *Journal of Interprofessional Care,* 27**,** 31-36.
LAURENSON, M. & BROCKLEHURST, H. 2011. Interprofessionalism, personalization and care provision. *British Journal of Community Nursing,* 16**,** 184-190.
LAWLIS, T. R., ANSON, J. & GREENFIELD, D. 2014. Barriers and enablers that influence sustainable interprofessional education: a literature review. *Journal of Interprofessional Care,* 28**,** 305-310.
MACDOUGALL, G., MATHEW, A., BROADHURST, V. & CHAMBERLAIN, S. 2001. An evaluation of an interprofessional palliative care education programme. *International Journal of Palliative Nursing,* 7**,** 24-29.
MACKAY, S. 2004. The role perception questionnaire (RPQ): a tool for assessing undergraduate students' perceptions of the role of other professions. *J Interprof Care,* 18**,** 289-302.
MARROCCO, G. F. 2014. Fostering significant learning in graduate nursing education. *J Nurs Educ,* 53**,** 177-9.
MCNAIR, R., BROWN, R., STONE, N. & SIMS, J. 2001. Rural interprofessional education: promoting teamwork in primary health care education and practice...Papers from the Australian College of Rural and Remote Medicine Inaugural Scientific Forum 'Steps Along the Pathway', Brisbane, Queensland, 10 June 2001. *Australian Journal of Rural Health,* 9**,** S19-26.
MENARD, P. & VARPIO, L. 2014. Selecting an interprofessional education model for a tertiary health care setting. *Journal of Interprofessional Care,* 28**,** 311-316.
MURDOCH, N. L., BOTTORFF, J. L. & MCCULLOUGH, D. 2013. Simulation education approaches to enhance collaborative healthcare: a best practices review. *Int J Nurs Educ Scholarsh,* 10.
NEWTON, C., BAINBRIDGE, L., BALL, V. A. & WOOD, V. I. 2013. Health Care Team Challenges: an international review and research agenda. *Journal of Interprofessional Care,* 27**,** 529-531.
NIEBUHR, V., NIEBUHR, B., TRUMBLE, J. & URBANI, M. J. 2014. Online faculty development for creating E-learning materials. *Educ Health (Abingdon),* 27**,** 255-61.
O'BRIEN, K., BONE, G., ZACK, E. & SOLOMON, P. 2008. HIV and rehabilitation: development of a conceptual framework for curriculum planning. *International Journal of Rehabilitation Research,* 31**,** 189-197.
O'CARROLL, V., MCSWIGGAN, L. & CAMPBELL, M. 2016. Health and social care professionals' attitudes to interprofessional working and interprofessional education: A literature review. *Journal of Interprofessional Care,* 30**,** 42-49.
OATES, M. & DAVIDSON, M. 2015. A critical appraisal of instruments to measure outcomes of interprofessional education. *Medical Education,* 49**,** 386-398.
PARKER, P. 2009. What should we assess in practice? *J Nurs Manag,* 17**,** 559-69.
PRIEST, H., SAWYER, A., ROBERTS, P. & RHODES, S. 2005. A survey of interprofessional education in communication skills in health care programmes in the UK. *Journal of Interprofessional Care,* 19**,** 236-250.
PULMAN, A., SCAMMELL, J. & MARTIN, M. 2009. Enabling interprofessional education: the role of technology to enhance learning. *Nurse Education Today,* 29**,** 232-239.
REEVES, S. 2001. A systematic review of the effects of interprofessional education on staff involved in the care of adults with mental health problems. *Journal of Psychiatric & Mental Health Nursing,* 8**,** 533-542.
REEVES, S. 2009. An overview of continuing interprofessional education. *Journal of Continuing Education in the Health Professions,* 29**,** 142-146.

REEVES, S., GOLDMAN, J., GILBERT, J., TEPPER, J., SILVER, I., SUTER, E. & ZWARENSTEIN, M. 2011. A scoping review to improve conceptual clarity of interprofessional interventions. *Journal of Interprofessional Care,* 25**,** 167-174.
REEVES, S., ZWARENSTEIN, M., GOLDMAN, J., BARR, H., FREETH, D., KOPPEL, I. & HAMMICK, M. 2010. The effectiveness of interprofessional education: key findings from a new systematic review. *Journal of Interprofessional Care,* 24**,** 230-241.
REYNOLDS, J., DILIBERTO, D., MANGHAM-JEFFERIES, L., ANSAH, E. K., LAL, S., MBAKILWA, H., BRUXVOORT, K., WEBSTER, J., VESTERGAARD, L. S., YEUNG, S., LESLIE, T., HUTCHINSON, E., REYBURN, H., LALLOO, D. G., SCHELLENBERG, D., CUNDILL, B., STAEDKE, S. G., WISEMAN, V., GOODMAN, C. & CHANDLER, C. I. 2014. The practice of 'doing' evaluation: lessons learned from nine complex intervention trials in action. *Implement Sci,* 9**,** 75.
SHOEMAKER, M. J., PLATKO, C. M., CLEGHORN, S. M. & BOOTH, A. 2014. Virtual patient care: an interprofessional education approach for physician assistant, physical therapy and occupational therapy students. *Journal of Interprofessional Care,* 28**,** 365-367.
SOCKALINGAM, S., TAN, A., HAWA, R., POLLEX, H., ABBEY, S. & HODGES, B. D. 2014. Interprofessional education for delirium care: a systematic review. *Journal of Interprofessional Care,* 28**,** 345-351.
SOLBERG, L. B., SOLBERG, L. M. & CARTER, C. S. 2015. Geriatric Care Boot Camp: An Interprofessional Education Program for Healthcare Professionals. *Journal of the American Geriatrics Society,* 63**,** 997-1001.
SUTER, E., GOLDMAN, J., MARTIMIANAKIS, T., CHATALALSINGH, C., DEMATTEO, D. J. & REEVES, S. 2013. The use of systems and organizational theories in the interprofessional field: Findings from a scoping review. *Journal of Interprofessional Care,* 27**,** 57-64.
THANNHAUSER, J., RUSSELL-MAYHEW, S. & SCOTT, C. 2010. Measures of interprofessional education and collaboration. *Journal of Interprofessional Care,* 24**,** 336-349.
THISTLETHWAITE, J. 2012. Interprofessional education: a review of context, learning and the research agenda. *Medical Education,* 46**,** 58-70.
THISTLETHWAITE, J., KUMAR, K., MORAN, M., SAUNDERS, R. & CARR, S. 2015. An exploratory review of pre-qualification interprofessional education evaluations. *J Interprof Care,* 29**,** 292-7.
THISTLETHWAITE, J. & MORAN, M. 2010. Learning outcomes for interprofessional education (IPE): Literature review and synthesis. *Journal of Interprofessional Care,* 24**,** 503-513.
VAN NORTWICK, S. S., LENDVAY, T. S., JENSEN, A. R., WRIGHT, A. S., HORVATH, K. D. & KIM, S. 2010. Methodologies for establishing validity in surgical simulation studies. *Surgery,* 147**,** 622-30.
VANLEIT, B., BANKS, L. & CRANDALL, C. 2012. Addressing complex multi-dimensional health problems using interprofessional education. *Medical Education,* 46**,** 525-526.
VELOSKI, J. J., FIELDS, S. K., BOEX, J. R. & BLANK, L. L. 2005. Measuring professionalism: a review of studies with instruments reported in the literature between 1982 and 2002. *Acad Med,* 80**,** 366-70.
WATERSTON, R. 2011. Interaction in online interprofessional education case discussions. *Journal of Interprofessional Care,* 25**,** 272-279.
WILSON, S. L., ROZENSKY, R. H. & WEISS, J. 2010. The Advisory Committee on Interdisciplinary Community-based Linkages and the Federal Role in Advocating for Interprofessional Education. *Journal of Allied Health,* 39**,** 210-215.
WRIGHT, A. & LINDQVIST, S. 2008. The development, outline and evaluation of the second level of an interprofessional learning programme -- listening to the students. *Journal of Interprofessional Care,* 22**,** 475-487.

ZHANG, C., THOMPSON, S. & MILLER, C. 2011. A Review of Simulation-Based Interprofessional Education. *Clinical Simulation in Nursing,* 7**,** e117-26.
