# Supplementary material for: Analysis of Six Reviews on the Quality of Instruments for the Evaluation of Interprofessional Education in German-Speaking Countries
Source: GMS J Med Educ. 2017 Aug 15;34(3):Doc36. doi: 10.3205/zma001113 (PMC5569985; doi:10.3205/zma001113)
Supplement: Short presentation of individual instruments [file JME-34-36-s-002.pdf]

**Readiness for Interprofessional Learning Scale (RIPLS)**

The "Readiness for Interprofessional Learning Scale" is one of the first questionnaires developed for the evaluation of interprofessional education (Parsell & Bligh, 1999). It consists of a total of 19 items and originally 3 scales for self-evaluation, which were developed by McFadyen et al. (2005) to 4 scales: Teamwork and Collaboration (9 items), Negative Professional Identity (3 items), Positive Professional Identity (4 items), Roles and Responsibilities (3 tems). The RIPLS is the most frequently used instrument for the evaluation of interprofessional education and is available in several languages (including German). On the whole, the use of the instrument is problematic in all languages since the factor structure cannot be depicted and it is not clear what the instrument actually measures (Mahler, Berger, & Reeves, 2015; Mahler et al., 2016).

**„University of the West of England Interprofessional Questionnaire" (UWEIPQ)**

The "University of the West of England Interprofessional Questionnaire" (Pollard, Miers, & Gilchrist, 2004, 2005) was created in the context of the development of a faculty program for interprofessional education (Pollard et al., 2004, 2005). It is a questionnaire for self-evaluation. It consists of 4 scales with a total of 35 questions: „Communication and Teamwork Scale" (9 items), „Interprofessional Learning Scale" (9 items), „Interprofessional Interaction Scale" (9 items), „Interprofessional Relationships Scale" (8 items). The scales can also be used individually. The "Interprofessional Relationships Scale" was not developed for evaluation of undergraduate education but for post-graduate application. The instrument is being used systematically for the evaluation of interprofessional teaching (for example Curtin University Perth) in some institutions. It has been translated into several languages (including German) and shows good psychometric values (Berger et al., 2013). The original factor structure could also be depicted in the German version (Mahler et al.,2016). The scales "Communication and Teamwork Scale", "Interprofessional Learning Scale" and "Interprofessional Interaction Scale" can be recommended for the evaluation of interprofessional training on Kirkpatrick Levels 1 and 2.

**Interprofessional Socialization and Valuing Scale (ISVS)**

The „Interprofessional Socialization and Valuing Scale" has been developed in Canada to measure the extent to which transformative learning (eg, socialization) has taken place, as measured by attitudes, beliefs, assumptions, and changes in the value of individuals (King, Shaw, Orchard, & Miller, 2010).

The scale is based on the assumption of a professional socialization and its necessary changes to achieve a successful interprofessional collaboration. The ISVS is a questionnaire

for self-evaluation and consists of originally 24 items. It has now been expanded to 34 items and consists of three scales: "Self-Perceived Ability to Work with Others" (11 items), "Value in Working with Others" (14 items) and "Comfort in Working with Others" (9 items ).

The instrument has so far been used successfully in different English-speaking countries and settings in education and shows good psychometric quality criteria in English. ISVS is currently only available in English. It is also used in Australia as a complement to the evaluation of skills in the area of the Capabilities Framework. It can be recommended for evaluations at Kirkpatrick levels 1 to 3.

Since the ISVS has not yet been validated in a German version, the authors have contacted the authors of the questionnaire and will, in close cooperation with them, translate the questionnaire into German and develop a validated German version.

The Working Group of the Committee therefore has the possibility to recommend the first instruments for the evaluation of interprofessional education.

**Team Climate Inventory (TCI)**

The "Team Climate Inventory" (TCI) was developed by Anderson and West and published in 1998 (Anderson & West, 1998). It was developed to measure the team function. It was used for interprofessional teams in outpatient and inpatient settings. The TCI consists of 38 items in the original version and is divided into four scales: "vision", "participative safety", "task orientation", and "support for innovation".

Yamani and colleagues (Yamani, Asgarimoqadam, Haghani, & Alavijeh, 2014) use the instrument to measure the effect of interprofessional training of health care teams on patient outcome. The extent to which the practical application of the instrument results in the basic training of the health professions seems to be currently open. It can be recommended for evaluations on the Kirkpatrick stage 4a (Barr et al., 2000), in particular also for continuing education. The instrument is currently not available in German.
